# Supplementary material for: Chitosan/Lactic Acid Systems: Liquid Crystalline Behavior, Rheological Properties, and Riboflavin Release In Vitro
Source: Int J Mol Sci. 2022 Oct 30;23(21):13207. doi: 10.3390/ijms232113207 (PMC9654581; doi:10.3390/ijms232113207)
Supplement: Supplementary file 1 [file ijms-23-13207-s001.zip › ijms-1973163-supplementary.pdf]

Supplementary Materials

Chitosan/Lactic Acid Systems: Liquid Crystalline Behavior, Rheological Properties, and Riboflavin Release In Vitro

Natalia M. Selivanova [1], Aliya I. Galeeva [1] and Yuriy G. Galyametdinov [1,2,*]

1 Department of Physical and Colloid Chemistry, Kazan National Research Technological University, Kazan 420015, Russia
2 Zavoisky Physical-Technical Institute, FRC Kazan Scientific Center of RAS, Kazan 420029, Russia
* Correspondence: yugal2002@mail.ru; Tel.: +78432314177

FTIR spectroscopy

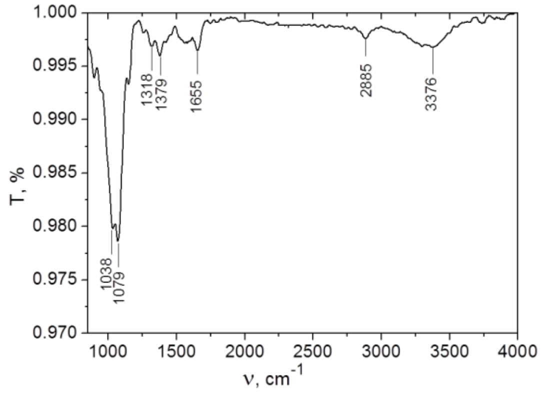

Figure S1 FTIR spectra of powdered chitosan.

In the FTIR spectrum of powdered chitosan a broad absorption band at 3376 cm^{-1} is attributed to the -OH and -NH stretching mode. The peak at 2885 cm^{-1} indicates the stretching of the CH bond in the methylene group. According to [1], the chitosan characteristic bands are the amide I band at 1650-1620 cm^{-1} (mainly C=O stretch) and amide II band at 1540-1520 cm^{-1} (C-N stretching coupled with N-H bending modes). In the spectrum of powdered chitosan, the bands at 1655 and 1562 cm^{-1} correspond to the C=O stretching and N-H bending mode, respectively. The band at 1318 cm^{-1} can be attributed to the symmetric bending of the protonated NH_3^+ group [2,3]. The absorption band at 1379 cm^{-1} corresponds to the OH bending mode. According to [3], the absorption in the frequency range of 900-1200 cm^{-1} indicates the C-O, C-N and C-C stretching modes at 1148, 1079, and 1938 cm^{-1}, respectively.

Rheological models

The experimental values were approximated using the microrheological models:

Newton Model $$\tau = \mu \cdot \dot{\gamma}\,, \quad \text{(S1)}$$

Bingham Model $$\tau = \tau_0 + \mu \cdot \dot{\gamma}\,, \quad \text{(S2)}$$

Power Law Model $$\tau = \mathrm{K} \cdot \dot{\gamma}^n, \quad \text{(S3)}$$

Casson Model $$\tau^{1/2} = \tau_0^{1/2} + (\mu \cdot \dot{\gamma})^{1/2}, \quad \text{(S4)}$$

Herschel –Bulkley Model $$\tau = \tau_0 + \mathrm{K} \cdot \dot{\gamma}^n\,, \quad \text{(S5)}$$

where τ – shear stress, Pa; μ – plastic viscosity, Pa·s; τ_0 – the yield stress, Pa; $\dot{\gamma}$ – shear rate, s^{-1}; K – consistency factor Pa·s^n; n – the flow index.

Table S1. Rheological characteristics of the systems at small $\dot{\gamma}$ = 0.07–7.5 s^{-1} and high $\dot{\gamma}$ = 7.5– 90 s^{-1} shear rates (Bingham model)

System	$\dot{\gamma}$ = 0.07–7.5 s^{-1}			$\dot{\gamma}$ = 7.5–90 s^{-1}		
	μ, mPa·s	τ_0, Pa	R, %	μ, mPa·s	τ_0, Pa	R, %
$C_{12}EO_4$/(Chit/LA) LCC	1305.0	1.98	88.8	349.7	12.2	94.7
Chit/LA Gel	812.0	0.23	78.5	331.0	6.77	89.1
Chit/LA LCC	16971.0	25.03	93.5	564.6	25.4	90.8

where R – correlation coefficient of experimental and calculated data,

$p<0.05$ vs. $C_{12}EO_4$

Table S2. Rheological characteristics of the systems at small $\dot{\gamma}$ = 0.07–7.5 s^{-1} and high $\dot{\gamma}$ = 7.5–90 s^{-1} shear rates (Power Law model)

System	$\dot{\gamma}$ = 0.07–7.5 s^{-1}			$\dot{\gamma}$ = 7.5–90 s^{-1}		
	K, mPa·s	n	R, %	K, mPa·s	n	R, %
$C_{12}EO_4$/(Chit/LA) LCC	4323.0	0.36	82.0	4757.0	0.48	94.8
Chit/LA Gel	1055.0	0.89	96.0	1511.0	0.71	97.1
Chit/LA LCC	45269.0	0.19	98.1	13328.0	0.35	92.7

where R – correlation coefficient of experimental and calculated data,

$p<0.05$ vs. $C_{12}EO_4$

Table S3. Rheological characteristics of the systems at small $\dot{\gamma}$ = 0.07–7.5 s^{-1} and high $\dot{\gamma}$ = 7.5–90 s^{-1} shear rates (Herschel –Bulkley model)

System	$\dot{\gamma}$ = 0.07–7.5 s^{-1}				$\dot{\gamma}$ = 7.5–90 s^{-1}			
	K, mPa·s	n	τ_0, Pa	R, %	K, mPa·s	n	τ_0, Pa	R, %
$C_{12}EO_4$/(Chit/LA) LCC	325.0	1.71	28.8	97.0	1532.0	0.70	7.1	98.0
Chit/LA Gel	1191.0	0.82	0.85	99.5	3495.0	0.55	- 5.02	99.8
Chit/LA LCC	30824.0	0.41	12.1	97.5	-	-	-	-

where R – correlation coefficient of experimental and calculated data,

$p<0.05$ vs. $C_{12}EO_4$

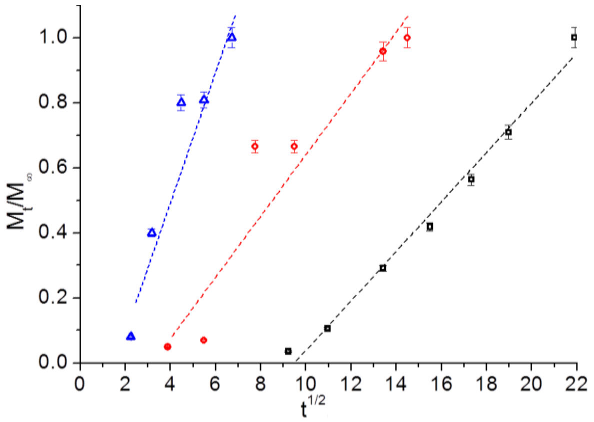

Figure S2 Release profiles for riboflavin from Chit/LA 15/85 wt.% ( ), Chit/LA 5/95 wt. % (C_{LA} = 2 wt.%) ( ), $C_{12}EO_4$/(Chit /LA) 50/50 wt.% ( ), plotted against square root of time.

References

1. Tien, C.Le.; Lacroix, M.; Ispas-Szabo, P.; Mateescu, M.A. N-acylated chitosan: hydrophobic matrices for controlled drug release. *J. Control. Release*, **2003**, *93*, 1–13. http://dx.doi.org/10.1016/S0168-3659(03)00327-4

2. Sinha, N.; Singh, B. K.; Dutta, P. K. Research on Antibacterial Screening and Drug Delivery using Chitosan-Stearic Acid Derivative, *J. Polym. Mater.*, **2017**, *34*, 11-20.
3. Bellamy, L. J. *The Infra-red Spectra of Complex Molecules*, 3rd. ed. New York: John Wiley Sons (Chapter. XIX), 1975, 433p. https://doi.org/10.1007/978-94-011-6017-9
